# Replacing Animal Fat with Gels of Psyllium Fiber and Combined Linseed Oil–Psyllium Fiber in Salamis: Impacts on Technological, Nutritional, Oxidative, and Sensory Properties

**DOI:** 10.3390/foods12132439

**Published:** 2023-06-21

**Authors:** Marcos Roberto Casarin Jovanovichs, Mariana Basso Pinton, Leticia Pereira Correa, Douglas Pedro, Carlos Augusto Mallmann, Roger Wagner, Alexandre José Cichoski, José Manuel Lorenzo, Alfredo Jorge Costa Teixeira, Paulo Cezar Bastianello Campagnol, Bibiana Alves dos Santos

**Affiliations:** 1 Departamento de Tecnologia e Ciência dos Alimentos, Universidade Federal de Santa Maria, Santa Maria 97105-900, Brazil; marcos.jovanovichs@iffarroupilha.edu.br (M.R.C.J.); mbpinton@gmail.com (M.B.P.); pereiracorreal@gmail.com (L.P.C.); douglas.pedro@iffarroupilha.edu.br (D.P.); mallmann@lamic.ufsm.br (C.A.M.); rogerwag@gmail.com (R.W.); cijoale@gmail.com (A.J.C.); paulocampagnol@gmail.com (P.C.B.C.); 2 Centro Tecnológico de la Carne de Galicia, Adva. Galicia n° 4, Parque Tecnológico de Galicia, San Cibrao das Viñas, 32900 Ourense, Spain; jmlorenzo@ceteca.net; 3 Área de Tecnología de los Alimentos, Facultad de Ciencias de Ourense, Universidade de Vigo, 32004 Ourense, Spain; 4 Laboratório para a Sustentabilidade e Tecnologia em Regiões de Montanha, Instituto Politécnico de Bragança, Campus de Santa Apolónia, 5300-253 Bragança, Portugal; teixeira@ipb.pt

**Keywords:** lipid reformulation, bioactive compounds, lipid oxidation, volatile compounds, sensory profile

## Abstract

This study produced two gels: one solely using psyllium fiber (GP) and another combining this fiber with linseed oil (GL+P). Both gels replaced 15% and 30% of the animal fat content of salamis. The objective was to evaluate the impact of this lipid reformulation on the technological, nutritional, oxidative, and sensory properties of the salamis. The lipid reformulation did not alter the evolution of pH and lactic acid bacteria during processing. The addition of GL+P did not interfere with the product’s drying process. However, replacing 30% of animal fat with the GP resulted in greater weight loss and a lower final Aw value. The lipid reformulation minimally affected the color of the salamis but significantly enhanced their nutritional profile. This improvement was marked by a decrease in fat content and an increase in protein. Specifically, in the samples with GL+P, there was a rise in linolenic acid content and a reduction in the n-6/n-3 PUFA ratio. Adding GP did not affect the salamis’ oxidative stability and sensory profile. However, substituting 30% of the animal fat with GL+P increased the TBARS values, and volatile compounds derived from lipid oxidation hampered the products’ sensory profiles. A reduction in these negative effects was observed when replacing 15% of the fat with GL+P, suggesting this to be the ideal dosage for balancing the nutritional benefits with maintaining the product’s oxidative stability.

## 1. Introduction

With its wide range of variations and styles, salami is a highly appreciated meat product, with consumption dating back centuries. However, despite its strong sensory quality, this product is rich in saturated fatty acids (SFA) and has a high ratio of polyunsaturated fatty acids (PUFAs) n-6/n-3, which may be harmful to human health [1]. A promising approach to confer healthier characteristics on this product is partially replacing animal fat with ingredients rich in bioactive compounds.

Linseed oil and psyllium fiber stand out among the compounds of great interest in this scenario. Linseed oil, which is full of omega-3 fatty acids, is celebrated for its benefits to cardiovascular, brain, and immune health and possesses antioxidants that combat oxidative stress and inflammation [2]. On the other hand, psyllium fiber, a soluble fiber with a history of use for its digestive properties, forms a gel in contact with water, aiding in regulating bowel movement and treating digestive disorders [3,4]. Additionally, this fiber has demonstrated the potential to reduce cholesterol levels, improve cardiovascular health, and regulate blood sugar levels. Psyllium fiber also has a prebiotic effect that can reduce the risk of certain digestive disorders [5]. However, incorporating these compounds into meat products is not without challenges, including issues of oxidative stability and sensory quality [6,7,8].

Considering these points, using linseed oil and psyllium fiber as gels could present a promising strategy. Gelation can mitigate some of the challenges associated with directly adding these compounds, thus balancing the nutritional benefits with the technological and sensory implications and potentially enhancing the quality and acceptance of meat products [6]. Thus, this approach should be investigated more deeply to maximize these compounds’ benefits in producing healthier salamis.

To date, the influence of applying psyllium fiber alone or mixed with linseed oil in a gel form on the quality of salamis has not been thoroughly investigated. Therefore, this study produced two types of gels: one using only psyllium fiber and another combining this fiber with linseed oil. These gels were incorporated into salamis, replacing 15% and 30% of their animal fat. The impact of this lipid reformulation on the technological, nutritional, oxidative, and sensory properties of the salamis was assessed.

## 2. Materials and Methods

### 2.1. Materials

Pork (moisture: 72.1%; fat: 2.5%; and protein: 22.35%) and pork backfat (moisture: 14.9%; fat: 82.1%; and protein: 4.15%) were acquired from local commerce, as along with condiments (garlic, sweet paprika, and spicy paprika), sodium chloride, glucose, and sucrose. The linseed oil and psyllium fiber were acquired from the company Giroil S.A. (Santo Angelo, Brazil), the tween 80 from the company Merck (São Paulo, Brazil), and the carrageenan from the company NovaProm (Guaiçara, Brazil). The chemical additives (sodium nitrite, sodium nitrate, and sodium ascorbate) were donated by the company Ibrac S.A. (Rio Claro, Brazil). The starter culture Floracarn SPX was donated by the company Chr. Hansen (Valinhos, Brazil). The remaining reagents and culture media used were acquired from Merck.

### 2.2. Preparation of the Gels of Psyllium Fiber and Combined Linseed Oil–psyllium Fiber

Two types of gels were formulated: the first, called GL+P, combined linseed oil and psyllium fiber, while the second, called GP, consisted only of psyllium fiber. The composition of the GL+P gel included 25% linseed oil, 1% tween 80, 4% carrageenan, 10% psyllium fiber, and 60% water. In preparing the GL+P gel, the oil phase was prepared separately by combining the linseed oil and the tween 80. Meanwhile, the aqueous phase was prepared by mixing carrageenan, psyllium fiber, and water. The oil phase was then gradually incorporated into the aqueous phase, resulting in the emulsion.

On the other hand, the GP gel was formulated using a mixture of 10% psyllium fiber, 4% carrageenan, and 86% water. The process started with mixing the carrageenan and the water, followed by the addition of the psyllium fiber. After homogenization, both gels were heated in a water bath until they reached an internal temperature of 80 °C. Finally, the gels were cooled and stored at 4 °C for 12 h before they were used in the salamis.

### 2.3. Preparation of the Salamis

The treatments evaluated in this study were as follows: Control, which was prepared using 80% pork and 20% pork backfat; P+L_15%_ and P+L_30%_, in which 15% and 30% of the pork backfat, respectively, was replaced with the combined linseed oil–psyllium fiber gel (GL+P); and P_15%_ and P_30%_, where 15% and 30% of the pork backfat, respectively, was replaced with the psyllium fiber gel (GP). The pork, pork backfat, and gels were ground with a 12 mm disc. The following ingredients were added relative to the meat mass in each treatment: sodium chloride (2.5%), sodium nitrite (0.015%), sodium nitrate (0.015%), glucose (0.5%), sucrose (0.5%), sodium ascorbate (0.25%), garlic (0.3%), sweet paprika (2.2%), spicy paprika (0.1%), and Floracarn SPX starter culture (0.025%). After mixing, the meat mass was stuffed into 60 mm diameter artificial collagen casings (approximately 15 cm long; 50 pieces of approximately 200 g each per treatment). The pieces were submerged in a 20% potassium sorbate solution bath and transferred to a maturing chamber with controlled temperature and relative humidity, where they remained for 28 days. The temperature and relative humidity (Tº/RH%) program followed the following sequence: on the first day, 25 °C/RH 95%; on the second day, 24 °C/93%; on the third day, 23 °C/90%; on the fourth day, 22 °C/85%; on the fifth day, 21 °C/80%; on the sixth day, 20 °C/75%; and from the seventh to the twenty-eighth day, 18 °C/75% [9].

### 2.4. Analyses Carried out during Processing

The parameters of pH, water activity (Aw), weight loss, thiobarbituric acid reactive substances (TBARS), and counts of lactic acid bacteria and micrococci were analyzed in triplicate (except for weight loss) during the salami manufacturing process (days 1, 7, 14, 21, and 28) as presented below.

#### 2.4.1. Physicochemical Analyses

The Aqualab 4TE device (Decagon, Pullman, DC, USA), properly calibrated according to the manufacturer’s guidelines, was used to measure the water activity (Aw). pH measurement was carried out using a digital pH meter with a glass electrode (Digimed—DM-23DC, São Paulo, SP, Brazil) at a ratio of 1:10 (sample to distilled water). Weight loss was calculated based on the difference between the ten pieces’ initial and final weight per treatment. The TBARS analysis was conducted following the method described by Bruna et al. [10], and the results were expressed in mg of malondialdehyde (MDA) per kg of sample.

#### 2.4.2. Microbiological Analyses

Samples of 25 g were obtained and homogenized in 225 mL of 0.1% peptone water, followed by decimal dilutions. To quantify the lactic acid bacteria, De Man Rogosa Sharpe agar was used and incubated at 37 °C for 48 h, while micrococci were evaluated using Mannitol Salt Agar under the same incubation conditions [11].

### 2.5. Analyses Carried out Immediately after Processing

After 28 days of processing, the salamis were removed from the maturation chamber. The collagen casing was removed, and the salamis were vacuum-packed and stored at 4 °C. The subsequent analyses, described below, were carried out in triplicate (except for color) during the first 7 days of this storage period.

#### 2.5.1. Chemical Composition

The lipid content was determined using the method described by Bligh and Dyer [12]. The contents of moisture (950.46), ash (920.153), and proteins (992.15) were quantified using the methodology of AOAC [13].

#### 2.5.2. Fatty Acid Profile

Lipids were extracted using the method described by Bligh and Dyer [12]. Subsequently, fatty acid methyl esters (FAMEs) were obtained by following the procedure outlined by Hartman and Lago [14]. The quantification of FAMEs occurred through gas chromatography using the GC-Agilent 7890B device (Agilent Technologies, SL, Madrid, Spain). The equipment had a flame ionization detector and an automatic sample injector HP 7683. The process used a fused silica capillary column SPTM-2560 (100 m, 0.25 mm internal diameter, 0.2 μm film thickness; Supelco Inc., Bellafonte, PA, USA). The chromatographic conditions followed the guidelines proposed by Domínguez et al. [15]. Each FAME was identified by comparing their retention times with authenticated standards. The results were expressed in g/100 g of sample. The atherogenicity (AI) and thrombogenicity (TI) indexes were calculated according to the equations proposed by Ulbricht and Southgate [16]:

(1) $$AI=\frac{C12:0+\left(4*C14:0\right)+C16:0}{\left(ƩPUFA\right)+\left(ƩMUFA\right)}$$

(2) $$TI=\frac{C14:0+C16:0+C18:0}{\left(0.5*ƩMUFA\right)+\left(0.5*n-6\right)+\left(3*n-3\right)+(\frac{n-3}{n-6})}$$

#### 2.5.3. Instrumental Color

The instrumental colors (L*, a*, and b*) of the salamis were evaluated with a Minolta CR-400 colorimeter (Minolta Sensing Inc. Konica, Japan) with spectral reflectance (calibration mode), a 10° viewing angle, D65 illuminant, and operation in the CIE (Lab*) system. For each treatment, 6 readings were taken at different points on 3 slices of 10 mm thickness. The global color difference (ΔE) was calculated by comparing the color differences of each modified treatment with the Control as per the methodology described by de Oliveira Faria et al. [17].

#### 2.5.4. Volatile Compounds

The solid-phase microextraction (SPME) technique was used to extract volatile compounds, as per the methodology developed by Domínguez et al. [18]. The headspace extractions, as well as the conditioning and injection of the samples, were conducted using the PAL RTC 120 automatic sampler (CTC Analytics AG, Zwingen, Switzerland). One gram of crushed sample was introduced into a 20 mL vial. To ensure a uniform temperature in the headspace, all samples were equilibrated at 37 °C for 15 min, which was the same temperature used in the extractions, extending the total time to 30 min. After extraction was complete, the fiber was inserted into the injection port of the 7890B gas chromatograph (Agilent Technologies, Santa Clara, CA, USA), equipped with an MS77 5977B mass detector. The separation of volatiles occurred in a DB-624 capillary column (30 m, 250 μm id, 1.4 μm film thickness; J&W Scientific, Folsom, CA, USA). Subsequently, the extracted compounds were identified by comparing their mass spectra with those contained in the NIST14 library (National Institute of Standards and Technology, Gaithersburg, USA), by comparison with authentic standards (Supelco, Bellefonte, PA, USA), and by calculating the relative retention index using a standard series of alkanes (C5–C14, Supelco 44585-U, Supelco, Bellefonte, PA, USA), confronting the data with the existing literature. The results were expressed in area units (AU) × 10^5^/g of the sample.

#### 2.5.5. Sensory Analysis

The sensory characteristics of the salamis were assessed through a descriptive sensory evaluation following the guidelines established by Wang et al. [19]. In the initial phase, 11 tasters were chosen based on their performance in triangular and basic taste recognition tests, as outlined in the method proposed by Stone and Sidel [20]. These tasters, aged between 18 and 45 years, were tasked with developing descriptor terms for sample characterization in a Free Choice Profiling session, wherein they also determined the reference standards for each sensory descriptor. Subsequently, these tasters underwent training in the selected references over three sessions, each lasting up to four hours.

A descriptive evaluation was conducted immediately post production to ascertain whether the lipid reformulation impacted the sensory profile of the salamis. The evaluation included the descriptors: rancid aroma, acid aroma, characteristic aroma, rancid taste, acid taste, characteristic taste, pleasant taste, juiciness, and characteristic color. All sensory analyses took place in private booths under fluorescent lighting. Salami samples, sliced into 2 mm pieces, were assigned three random numerical codes and presented to the panelists individually, following the complete balanced block approach as stated by Macfie et al. [21]. Each descriptor was rated using a 9 cm unstructured scale, where 1 represented ‘extremely disliked’ and 9 represented ‘extremely liked’.

### 2.6. Statistical Analysis

The experiment was repeated three times, and a generalized linear model was applied to analyze the physicochemical and microbiological data using the Xlstat software (Addinsoft, New York, NY, USA). In the model, treatments and processing days were considered a fixed effect, while repetitions were analyzed as a random effect. The interaction of the fixed factors in the analyses carried out during processing was also evaluated. The comparison of samples was performed through ANOVA, and the Tukey test was applied to compare the means (*p* < 0.05). The results of the sensory analysis were evaluated through the application of a GPA (generalized Procrustes analysis) map, which was generated from a matrix of 5 rows (5 treatments) and 88 columns (8 descriptors and 11 tasters).

## 3. Results and Discussion

### 3.1. Analyses Carried out during Processing

#### 3.1.1. pH, Aw, and Weight Loss

**Figure 1:**
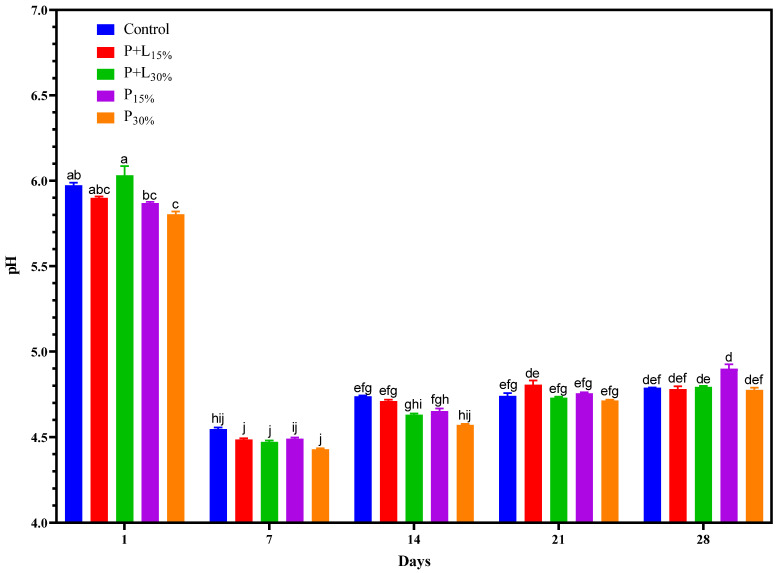
pH values during the processing of salamis that had undergone animal fat replacement with gels of psyllium fiber and combined linseed oil–psyllium fiber. Distinct letters demonstrate significant variations in Tukey’s analysis (*p* < 0.05). Error bars represent the standard error of the mean. Batches: Control: 20% pork back fat; P+L_15%_ and P+L_30%_: 15% and 30% substitution of pork back fat with combined linseed oil–psyllium fiber gel, respectively; P_15%_ and P_30%_: 15% and 30% substitution of pork back fat with gel of psyllium fiber, respectively.

A significant interaction between treatments and processing time for pH values was identified (Figure 1). On the first day of processing, the pH of all treatments remained between 5.8 to 6.0. The P_30%_ treatment had the lowest pH value (*p* < 0.05), which can be attributed to the acidic pH of psyllium fiber [22]. After 7 days of processing, a sharp drop in pH to around 4.5 was noticed in all treatments (*p* < 0.05). This decrease is consistent with the production of lactic acid by lactic acid bacteria during the processing of fermented meat products [23]. From the 14th day, there was an increase in pH, probably due to proteolytic reactions, which produce nitrogenous compounds that can increase pH [24,25]. The pH evolution in this study follows the standard dynamics reported in the literature for this type of meat product [23,24,26], suggesting that the reformulation did not adversely affect this parameter.

**Figure 2:**
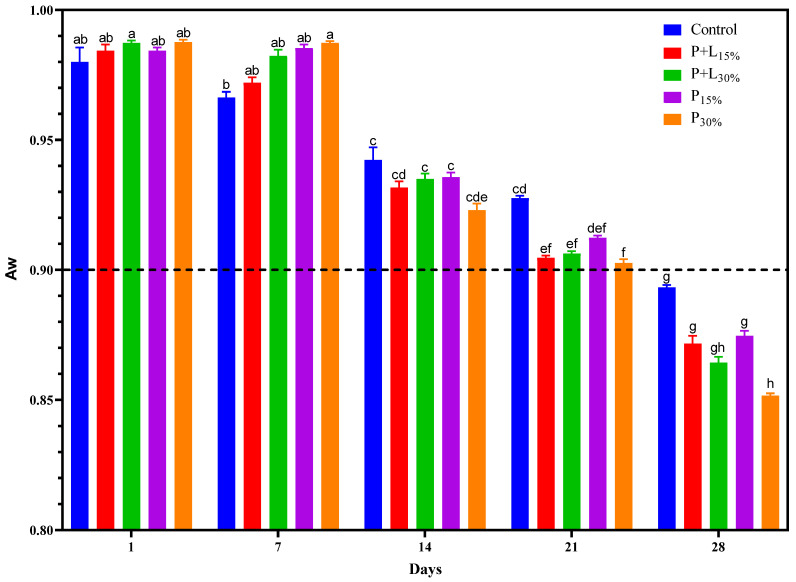
Aw values during the processing of salamis that had undergone animal fat replacement with gels of psyllium fiber and combined linseed oil–psyllium fiber. Distinct letters demonstrate significant variations in Tukey’s analysis (*p* < 0.05). Error bars represent the standard error of the mean. Batches: Control: 20% pork back fat; P+L_15%_ and P+L_30%_: 15% and 30% substitution of pork back fat with combined linseed oil–psyllium fiber gel, respectively; P_15%_ and P_30%_: 15% and 30% substitution of pork back fat with gel of psyllium fiber, respectively.

A significant interaction was detected regarding water activity (Aw) between the fixed factors, which in this study were the treatments and processing time (Figure 2). On the first day of processing, the Aw of all treatments was close to 0.98 (*p* > 0.05). From the 14th day, a decrease in Aw was observed. This reduction is associated with several factors in the salami manufacturing process. Firstly, the pH drop caused by lactic acid production by lactic acid bacteria can lead to the denaturation of the myofibrillar proteins, promoting water release [26]. Additionally, the decrease in relative humidity in the maturation chamber, where the products are stored, favors the product’s water loss [27]. After 28 days, all treatments had an Aw lower than 0.90. This condition benefits salamis’ microbiological safety and shelf life, as lower Aw levels limit the growth of pathogenic and spoilage microorganisms [28]. The P_30%_ treatment showed the lowest Aw value (*p* < 0.05) on the 28th day. This may be related to this treatment’s lower fat content (Table 1), as it may have facilitated more water loss [9]. However, the other treatments showed a final Aw similar (*p* > 0.05) to the Control, indicating that the reformulation did not compromise the Aw of the salamis, a critical factor for their conservation and safety.

**Figure 3:**
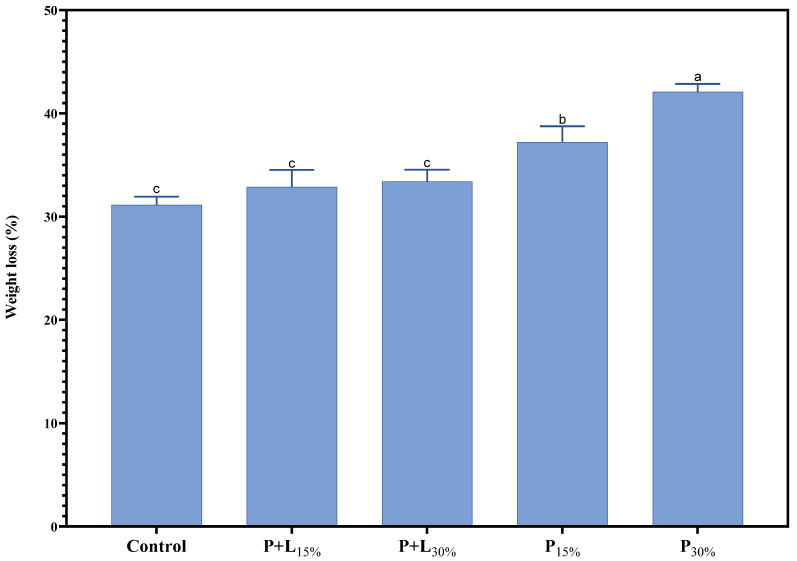
Weight loss of salamis that had undergone animal fat replacement with gels of psyllium fiber and combined linseed oil–psyllium fiber. Distinct letters demonstrate significant variations in Tukey’s analysis (*p* < 0.05). Error bars represent the standard error of the mean. Batches: Control: 20% pork back fat; P+L_15%_ and P+L_30%_: 15% and 30% substitution of pork back fat with combined linseed oil–psyllium fiber gel, respectively; P_15%_ and P_30%_: 15% and 30% substitution of pork back fat with gel of psyllium fiber, respectively.

Weight loss during salami processing varied from 31.1% to 42.1% (Figure 3). This weight loss is a common phenomenon in salami processing, being mainly attributed to the product’s dehydration during fermentation and maturation [29]. The weight loss range considered normal for salamis is between 30% and 35% [30,31]. The treatments containing GP+L gel (P+L_15%_ and P+L_30%_) showed weight loss similar (*p* > 0.05) to the Control. However, the treatments containing GP gel, especially P_30%_, showed significantly more weight loss. This additional weight loss may be associated with these treatments’ lower fat content (Table 2). Fat contains less moisture than lean meat. Therefore, salamis with less fat tend to lose more water during drying, resulting in greater weight loss [9]. The weight loss results align with those regarding Aw (Figure 2). The samples that exhibited more weight loss also exhibited lower Aw, which is expected, as water loss contributes to Aw reduction. This agreement reinforces the interpretation that the greater weight loss of the GP treatments is linked to their lower fat content.

#### 3.1.2. Lactic Acid Bacteria and Micrococci Counts

**Figure 4:**
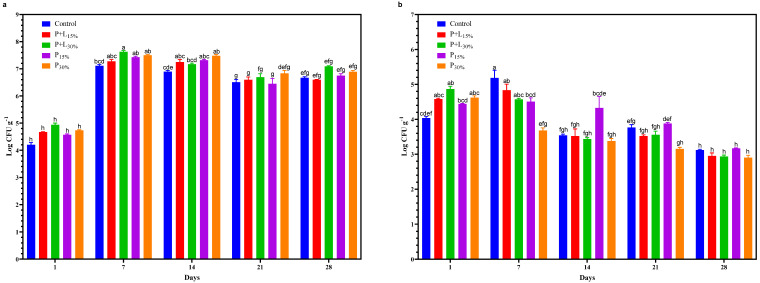
Lactic acid bacteria (**a**) and micrococci (**b**) counts of salamis that had undergone animal fat replacement with gels of psyllium fiber and combined linseed oil–psyllium fiber. Distinct letters demonstrate significant variations in Tukey’s analysis (*p* < 0.05). Error bars represent the standard error of the mean. Batches: Control: 20% pork back fat; P+L_15%_ and P+L_30%_: 15% and 30% substitution of pork back fat with combined linseed oil–psyllium fiber gel, respectively; P_15%_ and P_30%_: 15% and 30% substitution of pork back fat with gel of psyllium fiber, respectively.

A significant interaction between treatments and processing time was noted in the counts of lactic acid bacteria (Figure 4a) and micrococci (Figure 4b). Lactic acid bacteria demonstrated an increase to nearly 7 log CFU.g-1 after 7 days of processing and maintained this value until the end of the salami processing. This increase in the number of lactic acid bacteria during salami processing can be attributed to their ability to thrive in a low-oxygen environment and their resistance to the low pH typically found in salami. Furthermore, the metabolic activities of lactic acid bacteria contribute to the development of the organoleptic characteristics of the product, such as its taste and aroma, and are thus crucial to the fermentation process of salami [32]. Conversely, the population of micrococci displayed a slight growth in the initial 7 days of processing, followed by a decline in subsequent processing days. This trend can be explained by the reduced pH and oxygen concentration during salami processing. Micrococci are aerobic bacteria that are sensitive to acidity. Hence, the decreases in pH and oxygen concentration are limiting factors for their development [33]. The lipid reformulation exerted little influence on the evolution of lactic acid bacteria and micrococci during salami processing. The observed evolution of these microorganisms aligns with standard expectations for this type of meat product [34].

#### 3.1.3. TBARS

**Figure 5:**
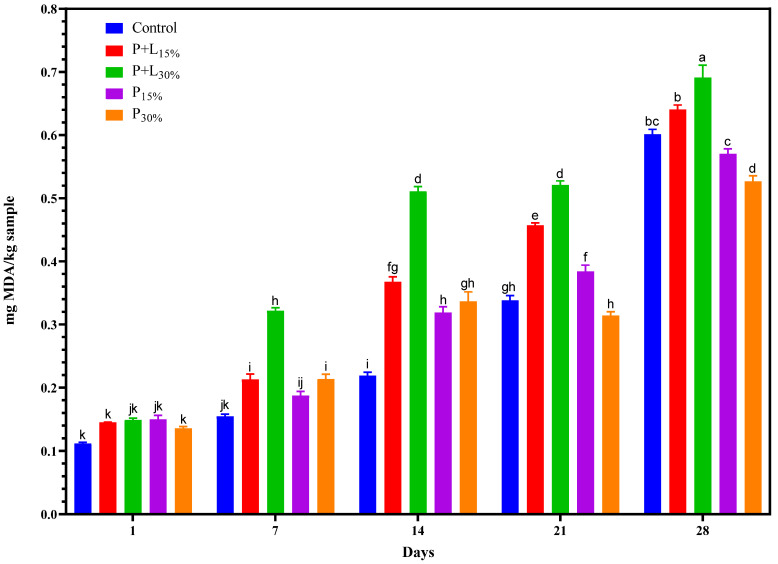
TBARS values during the processing of salamis that had undergone animal fat replacement with gels of psyllium fiber and combined linseed oil–psyllium fiber. Distinct letters demonstrate significant variations in Tukey’s analysis (*p* < 0.05). Error bars represent the standard error of the mean. Batches: Control: 20% pork back fat; P+L_15%_ and P+L_30%_: 15% and 30% substitution of pork back fat with combined linseed oil–psyllium fiber gel, respectively; P_15%_ and P_30%_: 15% and 30% substitution of pork back fat with gel of psyllium fiber, respectively.

The interaction between the fixed factors—namely, treatments and processing time—significantly influenced the TBARS values (Figure 5). On the first day of processing, the TBARS values were low, evidencing the good oxidative quality of the raw material and ingredients used.

The TBARS values increased during processing in all treatments, a normal occurrence in cured meat products. This increase results from lipid oxidation, which occurs as the unsaturated fatty acids present in the product interact with available oxygen during processing and storage. This oxidation leads to the formation of secondary compounds such as malondialdehyde, which are measured by the TBARS test [35].

From the seventh day, the treatment with the highest content of the GP+L gel (P+L_30%_) showed the largest increase in TBARS values. This can be attributed to increased polyunsaturated fatty acids (PUFAs), especially n-3 PUFAs (Table 2), due to the higher linseed oil content. PUFAs are more susceptible to oxidation due to the multiple double bonds in their structures, making them more reactive to oxygen, and therefore increasing TBARS values [27].

However, at the end of the processing period (28 days), the treatment that had undergone a 15% replacement of pork backfat with the GP+L gel (P+L_15%_) showed TBARS values similar to the Control (*p* > 0.05). This suggests that this replacement volume did not compromise the product’s oxidative stability. On the other hand, the treatments with the GP gel, especially P_30%_, showed lower TBARS values. This result could be related to these treatments’ lower fat content, reducing the substrate for oxidation and lowering TBARS values [27].

### 3.2. Analyses Performed Immediately after Processing

#### 3.2.1. Chemical Composition and Fatty Acid Profile

The chemical compositions of the salamis that had undergone the replacement of animal fat with linseed oil and/or psyllium fiber gels is presented in Table 1. The P_30%_ treatment showed a significantly lower moisture content (*p* < 0.05) when compared to the other treatments. This aligns with the Aw and weight loss results discussed earlier (Figure 2 and Figure 3). The protein contents of the modified treatments were significantly higher than that of the Control. This could be due to the protein concentration caused by the greater dehydration observed in these treatments (Figure 3). Reductions of 7.3%, 18.7%, 13.65%, and 25.7% in the fat content were observed in the P+L_15%_, P+L_30%_, P_15%_, and P_30%_ treatments, respectively, compared to the Control. Lastly, the ash contents of the treatments that underwent 30% fat replacement with the gels were significantly higher (*p* < 0.05). This could be attributed to the psyllium fiber, which is known to contain a significant amount of minerals [36].

**Table 1:** Chemical composition (%) of salamis that had undergone animal fat replacement with gels of psyllium fiber and combined linseed oil–psyllium fiber.

	Control	P+L_15%_	P+L_30%_	P_15%_	P_30%_	SEM	Sig.
Moisture	36.2 ^a^	34.3 ^a^	35.4 ^a^	35.1 ^ab^	34.1 ^b^	0.9	***
Protein	29.2 ^b^	31.5 ^a^	32.9 ^a^	32.4 ^a^	32.1 ^a^	1.2	***
Lipid	31.5 ^a^	29.2 ^ab^	25.6 ^cd^	27.2 ^bc^	23.4 ^d^	0.6	***
Ash	3.6 ^b^	4.4 ^b^	6.0 ^a^	4.4 ^b^	6.2 ^a^	0.1	***

Averages within the same line followed by the same letters did not show any significant difference (*p* > 0.05) according to Tukey’s test. Batches: Control: 20% pork back fat; P+L_15%_ and P+L_30%_: 15% and 30% substitution of pork back fat with combined linseed oil–psyllium fiber gel, respectively; P_15%_ and P_30%_: 15% and 30% substitution of pork back fat with gel of psyllium fiber, respectively. SEM: standard error of the mean. Sig: *** (*p* < 0.001).

The proposed reformulation also made the products’ fatty acid profile healthier (Table 2). The significant decrease in the total saturated fatty acid (SFA) contents of the modified treatments compared to the Control improved the nutritional quality of the product, as high levels of SFA in the diet may increase the risk of cardiovascular diseases [37]. The inclusion of linseed oil resulted in a significant increase in the linolenic acid content (C18:3n-3), leading to a reduction in the n-6/n-3 PUFA ratio. Maintaining an adequate balance between these fatty acids plays a role in preventing chronic diseases, such as cardiovascular and inflammatory diseases [38]. It is worth mentioning that conventional meat products usually possess high SFA contents and high n-6/n-3 PUFA ratios [39], which is a nutritional concern. Surprisingly, with only a 15% replacement of animal fat with the GP+L gel, a healthy n-6/n-3 PUFA [40] ratio was achieved.

**Table 2:** Fatty acid profile (mg/100 g sample) of salamis that had undergone animal fat replacement with gels of psyllium fiber and combined linseed oil–psyllium fiber.

	Control	P+L_15%_	P+L_30%_	P_15%_	P_30%_	SEM	Sig.
**C4:0**	14.5 ^a^	12.2 ^a^	8.1 ^a^	12.0 ^a^	10.0 ^a^	1.7	n.s
**C8:0**	6.5 ^a^	5.7 ^a^	4.0 ^a^	6.0 ^a^	4.8 ^a^	0.6	n.s
**C10:0**	15.0 ^a^	13.1 ^a^	10.6 ^a^	14.4 ^a^	12.2 ^a^	0.5	n.s
**C12:0**	16.4 ^a^	13.9 ^a^	11.3 ^a^	15.1 ^a^	13.1 ^a^	0.4	n.s
**C14:0**	321.1 ^a^	273.7 ^a^	219.9 ^a^	295.5 ^a^	255.1 ^a^	8.2	n.s
**C14:1n-5**	3.3 ^a^	2.9 ^ab^	2.4 ^b^	3.2 ^a^	2.9 ^ab^	0.1	***
**C15:0**	10.4 ^a^	9.0 ^a^	7.1 ^a^	9.2 ^a^	7.6 ^a^	0.3	n.s
**C16:0**	6417.2 ^a^	5587.6 ^b^	4538.8 ^c^	5877.6 ^b^	5091.2 ^bc^	175.8	***
**C16:1n-7**	678.7 ^a^	600.2 ^a^	480.3 ^a^	623.8 ^a^	552.6 ^a^	16.1	n.s
**C17:0**	63.9 ^a^	54.4 ^a^	42.8 ^a^	56.5 ^a^	47.2 ^a^	1.6	n.s
**C18:0**	2875.3 ^a^	2511.7 ^ab^	2133.7 ^b^	2676.2 ^ab^	2290.2 ^b^	86.3	***
**9t-C18:1**	18.2 ^a^	16.3 ^a^	11.9 ^a^	16.2 ^a^	12.1 ^a^	1.2	n.s
**11t-C18:1**	51.8 ^a^	48.8 ^a^	37.6 ^a^	53.6 ^a^	42.6 ^a^	4.2	n.s
**C18:1n-9**	13,255.9 ^a^	11,802.9 ^a^	9383.1 ^a^	11,924.0 ^a^	10,368.4 ^a^	377.1	n.s
**C18:1n-7**	1224.4 ^a^	1088.2 ^a^	853.5 ^a^	1114.5 ^a^	977.7 ^a^	27.5	n.s
**C18:2n-6**	3136.8 ^a^	2862.3 ^a^	2339.3 ^a^	2662.8 ^a^	2351.7 ^a^	104.6	n.s
**C18:3n-6**	5.9 ^a^	5.7 ^a^	4.3 ^a^	5.3 ^a^	4.5 ^a^	0.3	n.s
**C18:3n-3**	124.9 ^c^	710.9 ^b^	1197.5 ^a^	117.0 ^c^	94.4 ^c^	19.7	***
**9c,11t-C18:2 (CLA)**	31.0 ^a^	28.3 ^a^	21.3 ^a^	27.1 ^a^	23.5 ^a^	1.8	n.s
**C20:0**	68.3 ^a^	59.8 ^a^	50.3 ^a^	61.9 ^a^	54.1 ^a^	2.3	n.s
**C20:1n-9**	533.4 ^a^	454.3 ^a^	347.4 ^a^	469.3 ^a^	408.3 ^a^	16.1	n.s
**C20:2n-6**	265.7 ^a^	223.0 ^a^	159.7 ^a^	222.2 ^a^	195.8 ^a^	9.3	n.s
**C21:0**	10.6 ^a^	8.9 ^a^	7.6 ^a^	9.9 ^a^	8.3 ^a^	0.3	n.s
**C20:3n-6**	48.0 ^a^	41.6 ^a^	30.9 ^a^	40.4 ^a^	35.9 ^a^	2.4	n.s
**C20:4n-6**	126.3 ^a^	120.1 ^a^	109.3 ^a^	109.8 ^a^	124.0 ^a^	4.5	n.s
**C20:3n-3**	45.4 ^a^	38.7 ^a^	28.2 ^a^	37.8 ^a^	34.1 ^a^	1.5	n.s
**C20:5n-3**	10.3 ^a^	7.7 ^a^	4.8 ^a^	8.6 ^a^	7.0 ^a^	1.3	n.s
**C22:1n-9**	14.4 ^a^	12.5 ^a^	8.8 ^a^	12.2 ^a^	10.4 ^a^	0.7	n.s
**C22:2n-6**	10.5 ^a^	8.5 ^a^	6.0 ^a^	8.3 ^a^	7.1 ^a^	0.7	n.s
**C22:5n-3**	57.4 ^a^	51.5 ^a^	37.0 ^a^	47.3 ^a^	43.6 ^a^	4.3	n.s
**C22:6n-3**	11.8 ^a^	11.7 ^a^	9.1 ^a^	10.7 ^a^	10.6 ^a^	1.2	n.s
**∑SFA**	9820.0 ^a^	8550.7 ^b^	7034.7 ^c^	9034.8 ^ab^	7794.3 ^bc^	275.0	***
**∑MUFA**	15,780.4 ^a^	14,026.4 ^a^	11,125.4 ^b^	14,217.1 ^a^	12,375.5 ^ab^	441.2	***
**∑PUFA**	3874.5 ^ab^	4110.6 ^a^	3947.9 ^a^	3297.7 ^ab^	2932.91 ^b^	140.2	n.s
**PUFA/SFA**	0.39 ^bc^	0.48 ^ab^	0.56 ^a^	0.36 ^c^	0.37 ^c^	0.01	***
**∑n-3**	249.9 ^c^	820.7 ^b^	1276.7 ^a^	221.5 ^c^	189.9 ^c^	23.0	***
**∑n-6**	3624.5 ^a^	3289.8 ^a^	2671.2 ^b^	3076.1 ^a^	2742.9 ^ab^	122.5	***
**n-6/n-3**	14.5 ^a^	4.01 ^b^	2.09 ^b^	13.8 ^a^	14.4 ^a^	0.1	***
**AI**	0.39 ^ab^	0.36 ^bc^	0.36 ^c^	0.40 ^a^	0.40 ^a^	0.01	***
**TI**	0.92 ^a^	0.75 ^b^	0.64 ^c^	0.95 ^a^	0.93 ^a^	0.01	***

^a–c^ Mean values in the same row not followed by a common letter differ significantly (*p* < 0.05). Batches: Control: 20% pork back fat; P+L_15%_ and P+L_30%_: 15% and 30% substitution of pork back fat with combined linseed oil–psyllium fiber gel, respectively; P_15%_ and P_30%_: 15% and 30% substitution of pork back fat with gel of psyllium fiber, respectively. SEM: standard error of the mean. Sig.: significance: *** (*p* < 0.001); n.s. (not significant). SFA: saturated fatty acids; MUFA: monounsaturated fatty acids; PUFA: polyunsaturated fatty acids; n-6: omega-6; n-3: omega-3; AI: atherogenic index; TI: thrombogenic index.

Furthermore, the P+L_30%_ treatment showed an increase in the PUFA/SFA ratio and a decrease in the atherogenicity (AI) and thrombogenicity (TI) indices compared to the Control (*p* < 0.05). Thus, the changes in the lipid profile resulting from the addition of linseed oil indicate that the modified products are less likely to aggravate risk factors associated with the development of cardiovascular diseases [41] compared to the conventional product.

#### 3.2.2. Instrumental Color (L*, a*, and b*) and ΔE

**Figure 6:**
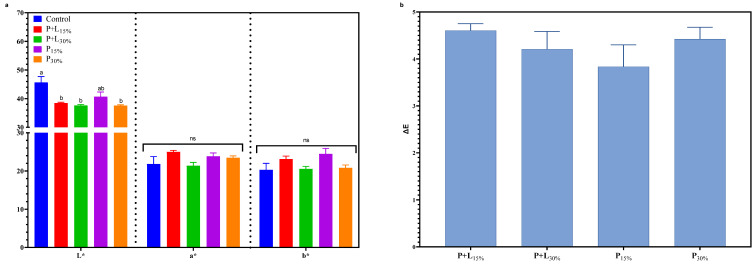
Instrumental color (L*, a*, and b*) (**a**) and ΔE values (**b**) of salamis that had undergone animal fat replacement with gels of psyllium fiber and combined linseed oil–psyllium fiber. Distinct letters demonstrate significant variations in Tukey’s analysis (*p* < 0.05). Error bars represent the standard error of the mean. Batches: Control: 20% pork back fat; P+L_15%_ and P+L_30%_: 15% and 30% substitution of pork back fat with combined linseed oil–psyllium fiber gel, respectively; P_15%_ and P_30%_: 15% and 30% substitution of pork back fat with gel of psyllium fiber, respectively. n.s.: not significant.

The instrumental color (L*, a*, and b*) and ΔE values are shown in Figure 6a and Figure 6b, respectively. No significant differences in the a* and b* values were found between the treatments. This result is highly relevant, as numerous studies have suggested that the replacement of fat by oils and fibers can modify these parameters, potentially impacting consumer acceptance [42,43]. However, the L* values were reduced in the modified treatments, except for P_15%_. This change in lightness could have occurred due to a series of factors. Firstly, it may be attributed to the lower fat content in the modified treatments, as fat tends to reflect more light than lean meat. Moreover, higher dehydration, a common characteristic in products with lower fat content, may have contributed to this reduction in lightness. Another factor that might have influenced this is the linseed oil’s and psyllium fiber’s inherent lightness incorporated into the treatments, which may be lower than pork fat’s, thus altering the L* value [27].

All modified treatments showed ΔE values lower than five compared to the Control (Figure 6b). This result suggests that the color difference observed in L* values, although statistically significant, is subtle and likely not visually perceptible by consumers. To clarify, ΔE is a metric used in color science to quantify the difference between two colors. This measure considers differences in L*, a*, and b* values, with a ΔE of below one generally imperceptible to the human eye. In contrast, a ΔE from one to five is typically considered a small to moderate difference. Therefore, a ΔE less than five indicates that the color changes in the reformulated salamis are likely imperceptible or only slightly noticeable to consumers [44].

#### 3.2.3. Volatile Compounds

**Figure 7:**
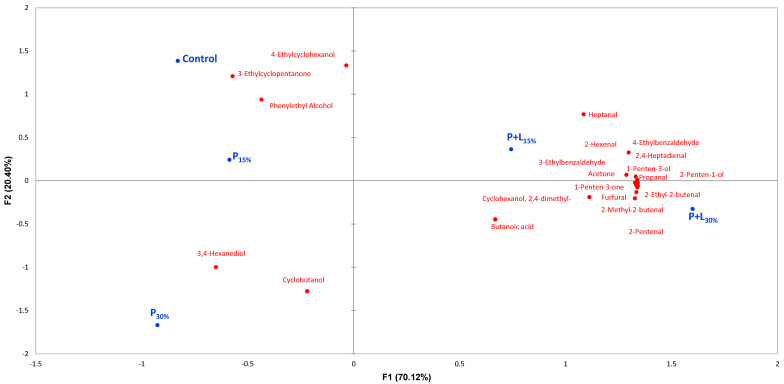
Principal component analysis (PCA) of the volatile compounds of salamis that had undergone animal fat replacement with gels of psyllium fiber and combined linseed oil–psyllium fiber. Batches: Control: 20% pork back fat; P+L_15%_ and P+L_30%_: 15% and 30% substitution of pork back fat with combined linseed oil–psyllium fiber gel, respectively; P_15%_ and P_30%_: 15% and 30% substitution of pork back fat with gel of psyllium fiber, respectively.

The analysis of the volatile compounds revealed 69 compounds, which were categorized based on their origin (Table 3). These compounds include five compounds derived from carbohydrate fermentation, ten from amino acid degradation, forty-seven from lipid oxidation, two from microbial catabolism, and five from spices. A principal component analysis (PCA) was performed using only the 21 compounds that showed significant differences among treatments (Table 3) to visually explore the effects of lipid reformulation (Figure 7). The first two principal components accounted for 90.52% of the total variation in the data, with PC1 representing 70.12% and PC2 contributing 20.04%. PC1 effectively discriminated samples containing linseed oil from those that did not. The P+L15% and P+L30% samples were positioned on the positive side of PC1, while the Control, P_15%_, and P_30%_ samples were placed on the negative side. The PCA results agree with the TBARS data (Figure 4), as the samples with linseed oil were strongly associated with 15 compounds derived from lipid oxidation. Some of these compounds, such as butanoic acid, 1-penten-3-ol, and heptanal, were present in large quantities (Table 3). The increase in volatile compounds from lipid oxidation in samples enriched with linseed oil can be attributed to the increased content of omega-3 fatty acids, as evidenced by the fatty acid profile analysis (Table 2). Omega-3 fatty acids are highly unsaturated and are therefore more susceptible to oxidation [45]. This lipid oxidation process begins with fatty acids reacting with oxygen, forming lipid peroxides. These peroxides, in turn, decompose, forming a variety of low molecular weight compounds, including aldehydes, ketones, and short-chain acids, which are the volatile compounds identified in the volatile profile of salamis. In contrast, the samples without linseed oil were characterized by only four lipid oxidation compounds, which were present in low concentrations (Table 3), and by one compound derived from amino acid degradation.

Lipid oxidation compounds play a complex role in the aroma of salami. On the one hand, they are often associated with the complexity and richness of the characteristic aroma of various meat products, playing a fundamental role in the sensory perception of these foods [23,46]. On the other hand, excessive lipid oxidation can lead to the development of off-flavors due to the formation of secondary oxidation products, which are generally considered undesirable [47]. These observations highlight the delicate balance that must be maintained in the production of salamis to ensure the product is safe and appealing to consumers. Including linseed oil in the formulation seems to influence this balance, as reflected in the volatile compounds profile. The increased presence of oxidation-prone omega-3 fatty acids resulted in a volatile profile with more lipid oxidation-derived compounds. However, precise control over the production process and the possible addition of antioxidants may be effective strategies in minimizing excessive oxidation and thus maintaining the quality and desirable aroma of the product. More studies are needed to investigate these possibilities and optimize the production of salamis enriched with omega-3 fatty acids.

#### 3.2.4. Sensory Analysis

**Figure 8:**
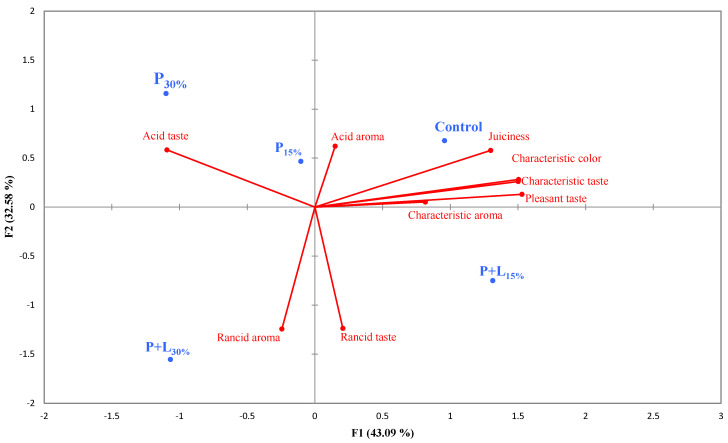
GPA map (generalized Procrustes analysis) of salamis that had undergone animal fat replacement with gels of psyllium fiber and combined linseed oil–psyllium fiber. Batches: Control: 20% pork back fat; P+L_15%_ and P+L_30%_: 15% and 30% substitution of pork back fat with combined linseed oil–psyllium fiber gel, respectively; P_15%_ and P_30%_: 15% and 30% substitution of pork back fat with gel of psyllium fiber, respectively.

The sensory analysis data were interpreted using a generalized Procrustes analysis (GPA) map (Figure 8). F1 explained 43.09% of the total variation in the data, while F2 explained 32.58%. The sensory analysis results correlated well with the TBARS data and volatile compounds. Notably, the tasters positioned the sample with the highest level of linseed oil (P+L_30%_) close to the attributes “rancid aroma” and “rancid taste”. This suggests that the increased presence of oxidation-prone omega-3 fatty acids may have contributed to the formation of volatile compounds associated with these undesirable sensory attributes. This phenomenon aligns with the findings of other studies investigating the influence of omega-3 fatty acid enrichment on meat products [48,49].

As expected, the Control sample was characterized by positive sensory attributes such as “juiciness”, “characteristic color”, “characteristic taste”, “pleasant taste “, and “characteristic aroma”. These attributes are associated with salami’s quality and expected taste, reinforcing the appropriateness of the production process used for the Control sample. The samples with a 15% substitution of pork fat with the gels (P_15%_ and P+L_15%_) were positioned close to the Control on the GPA map. This suggests that this substitution level does not seem to have caused major changes in the sensory profile of the salamis. Thus, lipid reformulation up to this level might be a viable strategy for producing salamis with an improved lipid profile without significantly affecting their sensory quality.

## 4. Conclusions

This study evaluated the effect of replacing 15% and 30% of animal fat with gels made from linseed oil and/or psyllium fiber on the quality of salamis. Both gels conferred nutritional benefits to the products, including reducing fat and increasing protein content. In addition, the gels with linseed oil provided a nutritional improvement in the fatty acid profile of the salamis due to the increase in linolenic acid. Lipid reformulation did not cause major changes in the color of the products, demonstrating that it is possible to obtain salamis with an improved nutritional profile without significantly compromising visual appearance. The substitution of animal fat with these gels significantly contributes to the overall health benefits of the salamis by reducing saturated fats and increasing dietary fiber and beneficial fatty acids, thus providing a healthier choice to the consumers.

The substitution of animal fat with the gel made only with psyllium fiber did not affect oxidative stability and had a minimal impact on the sensory profile of the products, especially at the 15% substitution level. On the other hand, the results of TBARS analysis, volatile compound analysis, and sensory profile analysis revealed that substituting animal fat with the gel with linseed oil should be carried out with caution to balance the nutritional benefits with maintaining the product’s oxidative stability. The 15% substitution level of animal fat with linseed oil and psyllium fiber gel was identified as the most efficient condition to achieve this balance, presenting favorable results regarding oxidative stability and sensory profile. However, further studies should be conducted to better understand the oxidative stability of the reformulated salamis and explore additional strategies, such as the incorporation of natural antioxidants, in order to further optimize the quality and safety of these products.

## Figures and Tables

**Table 3:** Volatile compounds (expressed as AU × 10^5^) of salamis that had undergone animal fat replacement with gels of psyllium fiber and combined linseed oil–psyllium fiber.

	Control	P+L_15%_	P+L_30%_	P_15%_	P_30%_	SEM	Sig.
**Carbohydrate fermentation**							
1-Butanol	0.38	0.27	0.22	0.45	0.39	0.02	n.s.
2,3-Butanediol	0.29	0.78	0.60	0.55	0.96	0.01	n.s.
Acetoin	2.30	2.54	1.46	1.54	1.61	0.21	n.s.
Acetone	1.00 ^b^	1.36 ^ab^	1.67 ^a^	0.99 ^b^	0.98 ^b^	0.11	***
2-Butanone	0.53	0.61	0.86	0.49	0.66	0.01	n.s.
**Amino acid degradation**							
3-Methyl-1-butanol	0.17	0.16	1.43	0.12	0.14	0.01	n.s.
Phenylethyl Alcohol	0.62 ^a^	0.22 ^b^	0.20 ^b^	0.15 ^b^	0.18 ^b^	0.01	***
3-Methylbutanal	1.35	1.63	1.92	1.34	1.80	0.15	n.s.
Furfural	0.05 ^b^	0.34 ^a^	0.53 ^a^	0.05 ^b^	0.03 ^b^	0.01	***
Benzaldehyde	6.67	7.47	6.76	5.77	6.56	0.23	n.s.
Benzeneacetaldehyde	2.03	2.62	2.09	2.20	2.19	0.15	n.s.
3-Methylthiophene	0.30	0.35	0.55	0.48	0.32	0.01	n.s.
2-Methyl-3-buten-2-ol	0.07	0.09	0.09	0.09	0.09	0.01	n.s.
Dimethyl trisulfide	0.04	0.02	0.03	0.08	0.06	0.01	n.s.
Dimethyl disulfide	0.07	0.05	0.07	0.11	0.09	0.01	n.s.
**Lipid oxidation**							
Butanoic acid	1.23 ^b^	2.41 ^a^	2.15 ^ab^	2.36 ^a^	1.90 ^ab^	0.16	***
Hexanoic acid	4.51	9.15	3.63	10.60	7.15	0.25	n.s.
1-Heptanol	3.25	3.52	1.95	3.32	2.60	0.12	n.s.
1-Hexanol	5.17	4.40	3.03	6.08	3.84	0.23	n.s.
1-Octanol	0.70	0.77	0.34	0.74	0.60	0.01	n.s.
1-Octen-3-ol	19.03	21.09	13.94	17.92	13.79	2.12	n.s.
1-Pentanol	8.98	7.41	5.91	10.50	9.13	1.15	n.s.
1-Penten-3-ol	4.19 ^c^	11.81 ^b^	18.63 ^a^	4.72 ^c^	2.96 ^c^	1.45	***
2-Penten-1-ol	0.17 ^b^	0.82 ^a^	1.22 ^a^	0.21 ^b^	0.16 ^b^	0.01	***
3-Pentanol	0.08	0.08	0.07	0.12	0.08	0.01	n.s.
4-Ethylcyclohexanol	0.25 ^a^	0.20 ^ab^	0.13 ^bc^	0.18 ^ab^	0.06 ^c^	0.01	***
Cyclobutanol	0.20 ^b^	0.22 ^b^	0.42 ^ab^	0.27 ^b^	0.72 ^a^	0.01	***
2,4-Dimethylcyclohexanol	0.18 ^b^	0.40 ^b^	5.89 ^a^	0.13 ^b^	0.06 ^b^	0.01	***
3,4-Hexanediol	0.13 ^b^	1.10 ^ab^	1.75 ^a^	0.19 ^b^	0.22 ^b^	0.01	***
2,4-Decadienal	0.65	0.55	0.40	0.73	0.27	0.01	n.s.
2,4-Heptadienal	0.22 ^b^	1.10 ^a^	1.38 ^a^	0.19 ^b^	0.13 ^b^	0.01	***
2,4-Nonadienal	0.78	1.19	0.55	1.01	0.71	0.02	n.s.
2-Ethyl-2-butenal	0.08 ^b^	1.87 ^ab^	2.76 ^a^	0.16 ^b^	0.15 ^b^	0.01	***
2-Methyl-2-butenal	1.09 ^b^	3.38 ^ab^	4.87 ^a^	1.86 ^b^	1.59 ^b^	0.16	***
2-Decenal	0.26	0.31	0.15	0.36	0.20	0.01	n.s.
2-Hexenal	0.47 ^bc^	0.80 ^ab^	0.99 ^a^	0.37 ^c^	0.21 ^c^	0.03	***
2-Ethyl-2-hexenal	0.12	0.21	0.13	0.24	0.32	0.01	n.s.
2-Nonenal	0.55	0.77	0.35	0.63	0.42	0.01	n.s.
2-Octenal	3.44	4.30	2.14	3.69	2.13	0.21	n.s.
2-Pentenal	0.40 ^c^	0.55 ^b^	2.16 ^a^	0.12 ^d^	0.09 ^d^	0.01	***
2-Propynal	0.36	0.43	0.24	0.37	0.22	0.01	n.s.
3-Ethylbenzaldehyde	0.39 ^b^	1.90 ^a^	1.85 ^a^	0.55 ^b^	0.35 ^b^	0.01	***
4-Ethylbenzaldehyde	0.28 ^b^	1.33 ^a^	1.30 ^a^	0.39 ^b^	0.25 ^b^	0.01	***
Butanal	0.21	0.19	0.17	0.22	0.17	0.01	n.s.
Heptanal	2.3 ^ab^	2.65 ^a^	2.77 ^a^	2.3 ^ab^	1.58 ^b^	0.41	***
Hexanal	16.18	14.47	12.91	16.69	15.10	3.12	n.s.
Nonanal	2.11	2.23	1.24	2.34	1.97	0.21	n.s.
Octanal	1.69	1.45	0.74	1.76	1.36	0.31	n.s.
Pentanal	4.22	3.61	2.72	4.58	3.64	0.62	n.s.
Propanal	0.65 ^b^	2.47 ^a^	3.25 ^a^	0.76 ^b^	0.61 ^b^	0.05	***
1-Penten-3-one	0.06 ^c^	0.22 ^b^	0.40 ^a^	0.05 ^c^	0.03 ^c^	0.01	***
2-Heptanone	2.37	2.40	1.78	3.02	3.16	0.25	n.s.
2-Octanone	0.13	0.17	0.11	0.16	0.27	0.01	n.s.
2-Pentanone	0.09	0.10	0.12	0.11	0.08	0.01	n.s.
3,5-Octadien-2-one	0.47	5.36	5.04	0.67	0.67	0.64	n.s.
3-Ethylcyclopentanone	0.14 ^a^	0.10 ^b^	0.06 ^c^	0.11 ^b^	0.05 ^c^	0.01	***
6-Methyl-5-hepten-2-one	0.30	0.52	0.36	0.40	0.50	0.01	n.s.
2-Methylfuran	0.12	0.29	0.34	0.29	0.27	0.01	n.s.
2-Pentylfuran	21.13	24.17	17.24	26.25	23.74	3.25	n.s.
2-Butylfuran	1.56	1.61	1.21	2.53	1.79	0.15	n.s.
2-Propylfuran	0.08	0.08	0.09	0.10	0.09	0.01	n.s.
Hexanoic acid, methyl ester	0.27	0.39	0.22	0.47	0.71	0.11	n.s.
**Microbial catabolism**							
Methyl formate	0.04	0.24	0.09	0.35	0.12	0.01	n.s.
2-methylpropanal	0.28	0.26	0.31	0.27	0.38	0.01	n.s.
**Species**							
Diallyl disulphide	0.38	0.46	0.32	0.33	0.33	0.01	n.s.
Methyl 2-propenyl disulfide	1.15	1.00	1.10	1.10	0.83	0.02	n.s.
o-Cymene	0.16	0.23	0.23	0.17	0.26	0.01	n.s.
p-Xylene	0.13	0.15	0.17	0.12	0.25	0.01	n.s.
Styrene	0.07	0.09	0.09	0.07	0.11	0.01	n.s.

^a–d^ Mean values in the same row not followed by a common letter differ significantly (*p* < 0.05). Batches: Control: 20% pork back fat; P+L_15%_ and P+L_30%_: 15% and 30% substitution of pork back fat with combined linseed oil–psyllium fiber gel, respectively; P_15%_ and P_30%_: 15% and 30% substitution of pork back fat with gel of psyllium fiber, respectively. SEM: Standard error of the mean. Sig: n.s. (not significant), *** (*p* < 0.001).

## Data Availability

The data used to support the findings of this study can be made available by the corresponding author upon request.
